# Incorporating patient perspectives in the development of a core outcome set for reproductive genetic carrier screening: a sequential systematic review

**DOI:** 10.1038/s41431-022-01090-1

**Published:** 2022-03-28

**Authors:** Ebony Richardson, Alison McEwen, Toby Newton-John, Ashley Crook, Chris Jacobs

**Affiliations:** Graduate School of Health, University of Technology Sydney, Sydney, NSW Australia

**Keywords:** Population screening, Genetic counselling, Genetic testing

## Abstract

There is currently no consensus on the key outcomes of reproductive genetic carrier screening (RGCS). This has led to a large amount of variability in approaches to research, limiting direct comparison and synthesis of findings. In a recently published systematic review of quantitative studies on RGCS, we found that few studies incorporated patient-reported outcomes. In response to this gap, we conducted a sequential systematic review of qualitative studies to identify outcomes exploring the patient experience of RGCS. In conjunction with the review of quantitative studies, these outcomes will be used to inform the development of a core outcome set. Text excerpts relevant to outcomes, including quotes and themes, were extracted verbatim and deductively coded as outcomes. We conducted a narrative synthesis to group outcomes within domains previously defined in our review of quantitative studies, and identify any new domains that were unique to qualitative studies. Seventy-eight outcomes were derived from qualitative studies and grouped into 19 outcome domains. Three new outcome domains were identified; ‘goals of pre- and post-test genetic counselling’, ‘acceptability of further testing and alternative reproductive options’, and ‘perceived utility of RGCS’. The identification of outcome domains that were not identified in quantitative studies indicates that outcomes reflecting the patient perspective may be under-represented in the quantitative literature on this topic. Further work should focus on ensuring that outcomes reflect the real world needs and concerns of patients in order to maximise translation of research findings into clinical practice.

## Introduction

Reproductive genetic carrier screening (RGCS) identifies individuals and couples with an increased risk of having a child affected by a recessive or X-linked condition, providing them with information to make informed reproductive choices. Research evaluating RGCS to date has spanned numerous countries with a variety of screening approaches, each working within different healthcare systems and societal settings. RGCS has quickly evolved from a targeted screening approach aimed at individuals with an increased a priori risk, to a widely available, pan-ethnic screening approach offered broadly to the general population. Such rapid advancements in an emerging field have in many instances outpaced research efforts aiming to evaluate the impact, benefits and harms of RGCS. Varied approaches to evaluating RGCS and a lack of consensus regarding the measurable outcomes of RGCS has resulted in heterogeneity across studies. As a result, it has been difficult to utilise existing research literature to inform evidence-based practice recommendations, which are considered the most rigorous approach to guiding clinical practice. Current practice recommendations supporting the offer of RGCS have instead relied on a consensus-based approach [1–3]. The Core Outcome Development for Carrier Screening (CODECS) Study aims to address this issue by developing a set of agreed outcomes in collaboration with key stakeholders including patients, health professionals, researchers and policy-makers; known as a core outcome set (COS) [4]. A COS is a minimum set of outcomes that should be measured and reported in all studies on a particular topic and can improve the overall quality, comparability and synthesis of research findings in a body of literature. While there are valuable insights to be gained from existing research efforts in this area, addressing the heterogeneity in research outcomes by developing a COS will ensure that a core set of evidence-based data will be available for future practice guidelines to draw upon.

The initial stage in the development of a COS involves a review of outcomes used in previous studies. The identified outcomes form a baseline ‘long list’ that is refined during a consensus process involving key stakeholders. A sequential systematic review of outcomes measured in studies on RGCS was conducted as the first step in the CODECS study. We divided this review according to the data types that were reported, in order to account for the different methodological approaches needed to extract outcomes from quantitative versus qualitative data. This article reports on the findings of the systematic review of outcomes in qualitative studies of RGCS, and compares these with our previously published systematic review of quantitative studies [5].

This review of qualitative studies contributes to the goal of applying a patient-centred approach to the development of a COS [6, 7]. Valuable insight can be derived from involving patients in the COS development process, and has been shown to enhance the relevance of the COS to patients as the end-users and lead to identification of outcomes that were not identified by professional groups alone [8, 9]. A key finding from our systematic review of quantitative studies was limited patient-reported outcome measures, and limited evidence of patient and public involvement in the design and conduct of included studies. As a result, the outcomes identified from quantitative literature predominantly reflect the priorities and perspectives of researchers and clinicians. Qualitative research methods provide rich insights into the patient perspective, and where an existing body of published qualitative literature is available, as is the case with RGCS, a systematic review of qualitative studies can be a valuable addition to the COS development process [10].

Therefore, this systematic review aims (i) to identify outcomes of importance to patients accessing population-based RGCS to consider for inclusion in a COS, and (ii) to compare the outcomes identified from the qualitative literature with those identified in our previous systematic review of outcomes in quantitative studies [5].

## Material and methods

This systematic review was registered with PROSPERO (CRD42019140793) and conducted per the Preferred Reporting Items for Systematic reviews and Meta-Analyses (PRISMA) statement and guidance from the Core Outcome Measurement in Effectiveness Trials (COMET) Initiative [6, 11]. We searched the Cochrane Database of Systematic Reviews, the Joanna Briggs Institute Systematic Reviews database, MEDLINE, and PROSPERO and found no similar systematic reviews undertaken or underway.

### Search strategy

This review utilised the same search strategy as a previously published systematic review of quantitative studies [5]. MEDLINE, EMBASE, CINAHL, and PsycINFO were searched on 1 July 2021 (illustrative search available in Supplementary File 1). Forward and backward searching was performed using reference lists of included publications and forward citation through Google Scholar.

### Study selection

All peer-reviewed published studies available in English that conducted qualitative research with individuals or couples who had accessed population-based RGCS for recessive or X-linked conditions were eligible for inclusion. For the purpose of this review, qualitative methods were defined as interviews or focus groups, and excluded interpretation of open-text responses from surveys. Studies exploring the perspectives of individuals with lived experience of conditions included in RGCS were excluded as the focus of this review was to identify process-specific outcomes from those undertaking RGCS. Title and abstract screening, then full-text screening was performed in 10% increments by two independent reviewers (ER and AC) until >85% interrater reliability was achieved, with the remainder reviewed by the primary reviewer (ER) only. Any disagreements were resolved through discussion, and where required, by input from a third reviewer (CJ).

### Quality assessment and risk of bias

Two reviewers (ER and CJ) scored the quality of studies included in both reviews using the QualSyst tool [12]. ‘Quality’ was defined in terms of the studies’ internal validity or the extent to which the design, conduct, and analyses minimised errors and biases. Assessment of bias was not used as grounds for exclusion but rather to give an overall summary of quality.

### Data extraction

Due to the large number of studies identified through our search, data extraction was conducted in 5-year increments until outcome saturation was reached. This methodology is suitable for situations where the size of the review would be unmanageable if conducted in full [6]. Outcome saturation was defined as the point at which no new unique outcomes were identified, and this occurred within two 5-year cycles (2010–2015, 2016–2020). This approach ensures that data extraction will continue until all relevant outcomes have been identified and prevent missing relevant outcomes from earlier research.

In the systematic review of qualitative studies, no studies were anticipated to have addressed outcomes specifically, as such our approach to data extraction was deductive and guided by methodology outlined in a previous systematic review [9]. Text excerpts relevant to outcomes, including quotes and themes developed by authors, were extracted verbatim using NVivo software [13]. A coding guideline was developed by the primary reviewer (ER) and piloted on 20% of studies with a second reviewer (CJ), and subsequently refined. The primary reviewer conducted the remainder of the data extraction and this was checked for agreement by a second and third reviewer (AC and CJ).

In the systematic review of quantitative studies, used as a comparison herein, we extracted all outcomes, and where supplied, their definition, measurement methods and time point using NVivo software [13]. A coding guide was developed and piloted by two reviewers (ER and AC) for 20% of studies to ensure consistency, with the remainder extracted by the primary reviewer (ER). The primary outcome was noted when specified, and basic study characteristics including study aim and demographics of participants were extracted in both reviews.

### Data analysis

Both quantitative and qualitative reviews utilised the same analysis method in order to permit direct comparison of the findings between both reviews. A narrative synthesis was conducted, utilising content analysis to facilitate frequency counts and tabulation of outcome domains represented in the qualitative literature.

The COMET taxonomy was used as a high-level framework to group outcomes, with the hierarchy consisting of ‘COMET core areas’ followed by ‘COMET outcome domains’ [14]. We elected not to define outcomes as adverse events/effects as there is currently no consensus definition for adverse outcomes in the context of genetic testing. Outcomes were grouped into more granular domains by ER, hereafter referred to as CODECS domains, and mapped to the COMET taxonomy. Definition of the domain and grouping of outcomes were developed iteratively with AC and taken to the study management group (CJ, AM, TNJ) for final review and consensus. Three new CODECS study domains were defined in addition to 24 domains previously defined in the quantitative review. Minor changes were made to the titles of five existing CODECS domains from the quantitative review to appropriately distinguish them from, or evolve them in line with new domains identified in the qualitative review, and two similar domains were pooled (Supplementary Material 2).

The number of studies reporting each outcome domain were compared between quantitative and qualitative studies to highlight areas of difference. We defined three categories (i) outcome domains that were seen only in qualitative studies (ii) outcomes domains that were seen in both qualitative and quantitative studies (iii) outcome domains that were seen only in quantitative studies. Absolute difference in proportion of studies reporting outcome domains is reported.

## Results

### Search strategy

**Fig. 1:**
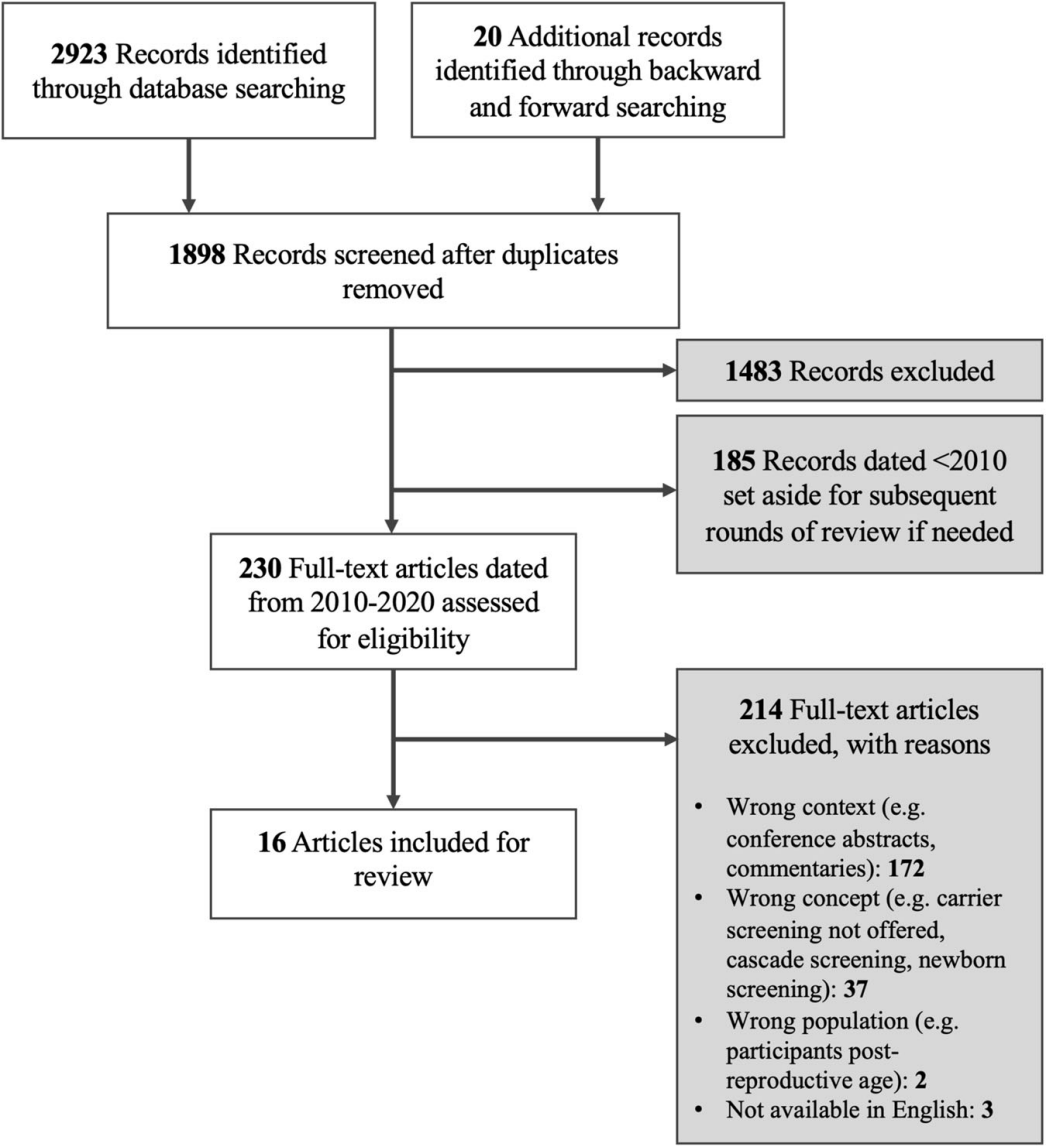
PRISMA Diagram.

Our literature search identified 2923 records. After de-duplication and title and abstract screening, 430 publications remained. The remaining publications were separated into 5-year periods, and 230 full-texts published between 2010 and 2020 were screened. Sixteen publications from 13 studies were eligible for inclusion in this review (Fig. 1) [15–30]. Six publications were from three mixed methods studies that were also included in our previous systematic review of quantitative studies [16, 18, 24, 25, 27, 30].

### Study characteristics

Study characteristics are summarised in Table 1. Eligible studies were from six countries, and incorporated a range of screening offers including targeted panels in founder populations (*n* = 5), haemoglobinopathies (*n* = 3), expanded carrier screening (*n* = 3), 3-gene panel (CF, FXS, SMA) (*n* = 2), and single gene screening (*n* = 2).

**Table 1:** Summary of Included Studies.

Year of Publication (*N* = 16)	Number of Studies
2020–2016	9
2010–2015	7
Country of Study (*N* = 13*)	
Australia	3
Canada	1
Israel	1
The Netherlands	3
UK	2
USA	3
Population^†^	
Average risk	5
Heterozygotes	7
Increased risk couples	5
Decliners of RGCS	2
Increased risk ethnic group before results available	1
RGCS results not disclosed (Dor Yesharim)	1
Intervention^†^	
Haemoglobinopathies	3
Targeted panel in founder population	5
Expanded carrier screening (ECS)	3
Cystic fibrosis (CF)	2
3-gene panel (CF, FXS, SMA)	2

Heterozygotes = one reproductive partner heterozygote for a recessive condition; Increased risk couples = female partner heterozygous for an X-linked condition, or both partners heterozygous for a recessive condition; Average risk = normal screening result with residual risk, Dor Yesharim = a confidential premarital screening program available in Jewish communities.

*16 publications from 13 studies.

^†^Some studies included multiple populations or interventions.

### Quality assessment and risk of bias

The mean quality assessment score was 0.67, with a range of 0.45–0.85 (maximum attainable score is 1) indicating a broad range of variability in the quality and risk of bias introduced across these qualitative studies. Of particular note, no studies incorporated reflexivity in the reporting of potential influences of the researcher or study methods on the findings Box 1. Few studies provided a description or justification of the theoretical framework or wider body of knowledge informing the study design and methods used. Scoring per study is available in Supplementary Material 3.

## Outcomes identified in qualitative studies of RGCS

The following results refer to the findings of the qualitative review only.

### Overview

**Fig. 2:**
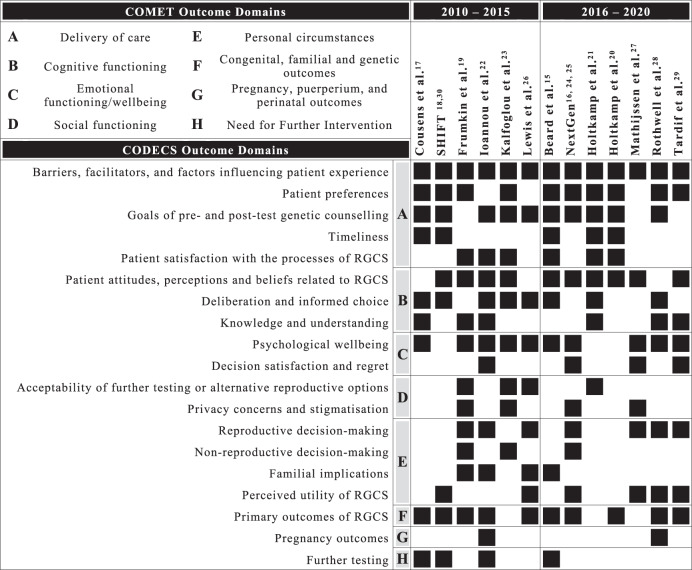
Outcomes domain across studies.

Seventy-eight outcomes were derived from qualitative studies included in this review, with a range of 7–32 outcomes per study and a median of 14. The majority of outcomes mapped to the COMET core areas of ‘life impact’ (*n* = 73, 94%), with the remainder mapping to ‘physiological/clinical’ (*n* = 3, 4%) and ‘resource use’ (*n* = 2, 3%). The highest number of outcomes were identified in the COMET domain of ‘delivery of care’ (*n* = 21, 27%), followed by emotional functioning/wellbeing (*n* = 19, 25%), personal circumstances (*n* = 16, 21%), cognitive functioning (*n* = 14, 18%), social functioning (*n* = 3, 4%), need for further intervention (*n* = 2, 3%), pregnancy, puerperium, and perinatal outcomes (*n* = 2, 3%), and congenital, familial and genetic outcomes (*n* = 1, <1%). At the most granular level, outcomes were grouped into 19 CODECS domains, with distributions of outcomes across studies shown in Fig. 2.

### Delivery of care

Twenty-one outcomes related to the COMET domain ‘delivery of care’ and were grouped in the CODECS domains ‘barriers, facilitators and factors influencing patient experience’, ‘patient preferences’, ‘goals of pre- and post-test genetic counselling, ‘timeliness’, and ‘patient satisfaction with the process of RGCS’. All studies included quotes or themes related to ‘barriers, facilitators and factors influencing patient experience’ of RGCS, this was the only CODECS domain that was uniformly represented across all included studies. These outcomes were most frequently related to barriers and facilitators to uptake of RGCS, followed by factors influencing emotional reactions and psychological wellbeing of patients.

Of the outcomes that mapped to ‘delivery of care’, the greatest number of outcomes were grouped in the CODECS domain ‘goals of pre- and post-test genetic counselling’ which was identified in 10 studies. Quotes and themes that informed this domain reflect patient needs at pre-test and post-test timepoints and how well these are met, and can be broadly categorised into two groups. First, outcomes related to information needs; including whether sufficient information was provided to meet patient needs, whether the timing and method of information provision promoted understanding, and whether the information provided supported informed decision-making. Second, outcomes related to providers of genetic counselling; including whether the provider was accessible, knowledgeable, presented RGCS as a choice, and was empathetic.

### Emotional functioning/well-being

Nineteen outcomes related to the COMET domain ‘emotional functioning/wellbeing’ and were grouped in the CODECS domains ‘psychological wellbeing’ and ‘decision satisfaction and regret’. Outcomes were associated with four timepoints; waiting for results, receiving results, undergoing further testing and prenatal decision-making, and long-term. The majority of these outcomes were in the CODECS domain ‘psychological wellbeing’ and were identified in 10 studies. A variety of emotional reactions were captured in the outcomes derived from included studies and where possible were extracted verbatim to demonstrate the different terminology used by patients in order to gain a better understanding of meaningful psychological outcomes to assess in this area. A range of illustrative words were used by patients to relay their emotional experience including anxiety, distress, fear, grief, relief, sadness, shock, sorrow, stress and worry. The most frequent psychological outcome was ‘shock’ (*n* = 6), followed by ‘anxiety’ (*n* = 4), and ‘relief’ (*n* = 4). Many of the psychological outcomes identified in qualitative studies were also identified in quantitative studies, with the exception of grief which was unique to qualitative studies. Specific outcomes that relate to the experience of pregnancy following an increased risk result were also identified, including detachment from a current pregnancy, difficulty feeling happy to fall pregnant, and loss of spontaneity around conception.

Factors that influenced emotional wellbeing could be deduced from some studies and included feeling supported by a genetic counsellor [15], the strength of the couple’s relationship and coping strategies [27], having sufficient pre-test information [17], and having a low pre-test perceived risk of an increased risk result [15, 21, 22, 28, 29].

### Personal circumstances

Sixteen outcomes related to the COMET domain ‘personal circumstances’ and were grouped in the CODECS domains ‘reproductive decision-making’, ‘non-reproductive decision-making’, ‘familial implications, and ‘perceived utility of RGCS’. Outcomes in this domain related to how the personal circumstances of the individual, couple, or wider family were impacted by RGCS. How the results of RGCS influenced reproductive decision making was most frequently represented, being identified in 7 studies. Six studies included quotes or themes that reflected patients perceived utility of RGCS, which was characterised by two aspects. First, utility was defined by the timeliness of results, with emphasis being placed on earlier results or preconception offers in order to allow sufficient time for consideration and decision making. Second, utility was reflected by patients’ sense of confidence or empowerment in their reproductive decisions.

## Findings of the sequential review

The following results refer to the findings across all studies, both quantitative and qualitative, and provides a comparison of the outcomes that were identified.

### Distribution of studies

Across both quantitative and qualitative systematic reviews of studies published between 2010 and 2020, we identified 77 publications from 57 studies. These included 14 publications from 4 mixed methods studies, 9 publications from 9 qualitative studies, and 54 publications from 44 quantitative studies.

### Outcomes and domains

**Fig. 3:**
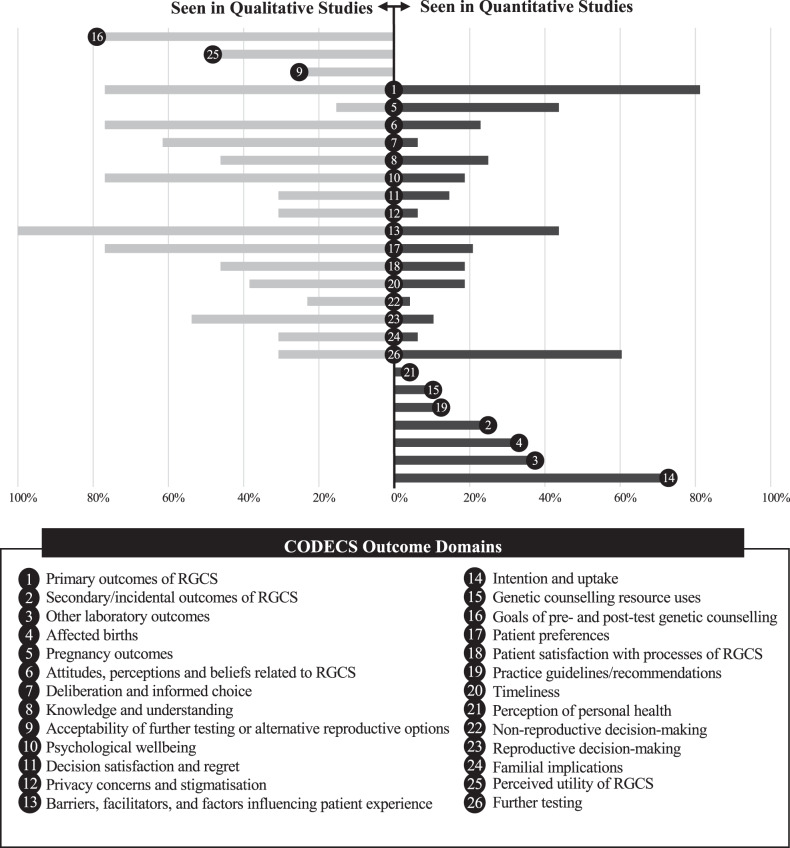
Comparison of the proportion of studies reporting CODECS outcome domains. Left – outcome domains that were seen only in qualitative studies. Central – outcomes domains that were seen in both qualitative and quantitative studies. Right – outcome domains that were only seen in quantitative studies.

We identified 163 outcomes grouped into 26 CODECS domains. Sixteen domains were represented in both quantitative and qualitative studies, 7 domains were identified in quantitative studies only, and 3 domains were identified in qualitative studies only (Fig. 3). The three CODECS domains that were newly identified in the qualitative review were ‘goals of pre- and post-test genetic counselling’, ‘acceptability of further testing and alternative reproductive options’, and ‘perceived utility of RGCS’.

Box 1 Comparison of key findings from sequential systematic reviews of quantitative and qualitative studies

## Discussion

This systematic review of qualitative studies identified outcomes of importance to patients accessing RGCS. The outcomes identified provide rich insights into the perspectives and needs of patients in relation to RGCS, and are valuable additions to the ‘long list’ of outcomes being considered for inclusion in a COS. Importantly, this review identified outcomes that were not identified in a previous published systematic review of outcomes measured in quantitative studies [5], with 3 new outcome domains being defined.

The first CODECS domain newly identified in the qualitative review was ‘goals of pre- and post-test genetic counselling’. This domain captures outcomes related to the patient experience of pre- and post-test interactions with their health providers. Genetic counselling in this context can be performed by a range of health professionals, which may include genetic counsellors as specialised providers but often involves a range of other non-genetic health professionals. Outcomes in this domain reflect recognised goals of genetic counselling as defined by the Human Genetics Society of Australasia and the National Society of Genetic Counsellors (USA), including the interpretation of family and medical history to assess chance of disease occurrence, education and counselling to promote informed choice, and support to encourage the best possible adjustment to genetic information [31, 32]. These outcomes also reflect criteria used to assess genetic screening programs broadly, such as aspects of voluntariness, accessibility, and the provision of good quality, comprehensible and balanced information [33, 34]. The overlap of outcomes we identified, with these goals and criteria, highlights that these are not only outcomes that are needed to informed evidence-based practice recommendations at a procedural level but also practical considerations of importance to patients. Many of the direct quotes that informed outcomes in this domain reflected perceived inadequacies of the RGCS programs, for instance indicating that information needs hadn’t been met, suggesting that there is room for improvement in the delivery of RGCS programs. There is a need for outcomes that reflect the goals of pre- and post-test genetic counselling to ensure that we capture whether patient needs are being met.

The second CODECS domain newly identified in the qualitative review was ‘acceptability of further testing and alternative reproductive options’. This domain captures outcomes related to patients’ perspectives on prenatal diagnosis, termination of pregnancy, and preimplantation genetic diagnosis. Personal preferences, religious and societal views, and practical difficulties were discussed in relation to these options. These concepts reflect contextual considerations around the implications of RGCS that are often not explored. Acceptability is a multi-faceted construct that reflects the extent to which people receiving a healthcare intervention consider it to be appropriate, and which encompasses both anticipated (prospective) and experienced (retrospective) aspects [35]. Acceptability as a concept is represented in quantitative studies of RGCS largely via uptake, with the assumption that if services are utilised then they are acceptable to patients. However, this does not account for the complex processes that can surround acceptability, nor does it consider retrospective acceptability once patients have lived experience of the process. It is evident from the identification of acceptability relating specifically to further testing and alternative reproductive options that acceptability beyond uptake would be valuable to explore in this setting. It is also important to recognise that all healthcare decisions are made within a societal context, and external influences can have significant impacts on the patient experience. The social impacts of RGCS are under-explored and measuring outcomes related to the social context in which decisions around RGCS, further testing, and reproductive decisions are made warrants further investigation, especially as RGCS becomes increasingly accessible to the general population.

The final CODECS domain newly identified in the qualitative review was, ‘perceived utility of RGCS’. This domain captures outcomes related to patients perceptions of the impact of RGCS and how they utilised the results. Two components of utility were identified from qualitative studies; that results instilled a sense of confidence and empowerment related to reproductive decisions, and that utility was dependent upon results being available in a timely manner that allowed for consideration and decision-making. When considering utility, we must consider the aims of RGCS programs and how these can be operationalised as measurable outcomes. Whilst there is no consensus definition of the primary aim of RGCS, there are two aims that are often stated; to support reproductive autonomy through the provision of information regarding reproductive risk in order to allow couples’ to make informed decisions regarding family planning, and to reduce the incidence of genetic conditions [36, 37]. In our review of quantitative literature, utility was reflected in outcomes such as reduced birth rate, as well as intended and actual reproductive behaviours of those identified as increased risk. Timeliness was also represented in some quantitative studies, with utility being compromised if there was insufficient time for deliberation and decision-making. Whilst in our review of qualitative studies, we identified reproductive empowerment and timeliness as two components of patients’ perceived utility of RGCS. Combining the findings of these sequential systematic reviews of outcomes in RGCS, it is evident that a consensus definition of the clinical utility of RGCS would be valuable and should consider aspects of empowerment, timing and reproductive decisions in order to reflect the clinical utility of RGCS in future studies.

A high degree of outcome heterogeneity was identified in the domain of ‘psychological wellbeing’ in our review of quantitative studies, with variability in the selection of outcomes and measurement methods that has limited the ability to compare psychological wellbeing across RGCS programs and hindered clear demonstration of benefits or potential harms. In this review we anticipated that direct quotations and themes from qualitative studies would provide greater insight into appropriate psychological outcomes to consider. Most notable was the outcome of grief, which was not seen in quantitative studies, but was represented in a number of qualitative studies. Grief was reflected in terminology such as ‘sorrow’ and ‘great sadness’, and encompassed multiple timepoints including the post-test period when individuals were identified as increased risk, when undertaking further testing and making decisions about a current pregnancy, and long-term when working towards a healthy pregnancy. We can look to examples from obstetrics and fertility settings, where work has been done in assessing grief, to consider appropriate measures that could be utilised in studies on RGCS. Grief related to pregnancy loss including early miscarriages through to later term and postnatal losses, in addition to grief related to unsuccessful fertility treatments, have similarities to the journey of increased risk couples identified through carrier screening, whereby the journey to a healthy pregnancy may take a more difficult and medicalised path than natural conceptions. Validated measurement tools are available to assess perinatal grief and may be suitable to adapt to the carrier screening setting [38]. It is important to acknowledge that the goal of assessing grief in this setting relates to potential adverse outcomes of RGCS, a rigorous understanding of which is needed to inform evidence-based practice recommendations and ensure that appropriate supports are in place for those that may experience complex grief following RGCS.

Whilst we did not categorise outcomes as adverse in this review, it was evident from the literature that many of the verbatim excerpts reflected perceived inadequacies of RGCS programs. For example, many of the goals of pre-test counselling were not met, most often in regards to information provision but also encompassing aspects such as timing of service delivery, presenting RGCS as a choice, feeling that decision-making was informed and that implications of testing were understood. Routinisation of genetic testing and whether the goal of truly informed choice is achievable has been explored in the setting of prenatal testing, and likely will have relevance for RGCS as it becomes a mainstay of preconception and early prenatal care [39–41]. Negative experiences in the form of grief and regret were also identified. There is currently no consensus definition of adverse outcomes from genetic testing. Identification of these is crucial for ensuring patient wellbeing, and initial analysis would suggest that adverse outcomes can be minimised in various ways. For example, regret may be related to feeling uninformed or that testing was not voluntary, leading to a lack of ownership over the decision to have testing. The nature of RGCS means that grief is likely to play a role in many cases, however complex grief may be minimised by providing appropriate supports throughout the process and identifying those at-risk that may require additional resources. Definition of adverse outcomes will be an important element to consider in the development of a COS. It is crucial to understand if individuals who undertake RGCS are at risk of complicated or prolonged grief or have unmet genetic counselling needs in order to consider how this can be minimised and used to inform implementation of RGCS.

The risk of bias associated with qualitative studies in this review identified some areas of consideration when interpreting findings. Overall mean quality assessment scores indicate potential risk of bias in this body of literature, consistent with findings in our review of quantitative studies. Of particular note, no studies reported a reflexive account of potential influences of the researcher or study methods on their findings. There are varied arguments for the necessity of reflexivity in qualitative research, however it is broadly agreed that consideration of researcher influence is an important element of rigor [42, 43]. In this context, the absence of reflexivity limits the transparency of these studies. Crucial points at which bias may be introduced in research include definition of study aims, interview guides and questions asked of participants, and interpretation of themes from the resulting data. If not accounted for, the perspective of researchers and clinicians involved in the study may skew the data towards their goals. We acknowledge that many studies incorporated aspects such as co-coding into their study design which illustrate that researcher positioning and influence were considered, however, have not been transparently reported. Practical limitations such as word counts in journal articles are also acknowledged, however in accordance with the COREQ guidelines for reporting of qualitative studies we suggest that a concise statement to summarise that reflexivity has been considered should be a minimum expectation in the reporting of future qualitative studies [44].

Using qualitative methods to explore patient experience can be a valuable tool to identify outcomes that are relevant to patients and ensure that research findings have direct translational impact on clinical practice [45]. Of the qualitative studies that were reviewed, only one paper by Lewis et al. explicitly aimed to identify outcomes [26]. This study applied a grounded theory approach to interviews conducted with individuals who had undergone RGCS, and identified reproductive empowerment as the main motivator and outcome of carrier screening. As previously mentioned, quantitative studies also lacked involvement of patients in the definition of research outcomes, with no reports of patients involved in the design of studies and selection of outcomes, and few studies utilised patient-reported outcomes. This limited representation of the patient perspective in regards to outcomes that are relevant and informative in this setting, across both quantitative and qualitative studies of RGCS, indicates that the real world needs and concerns of patients undertaking RGCS may be under-represented in current literature. Despite Lewis et al. providing the first example of a qualitative study aimed at identifying a key outcome of RGCS, no subsequent published studies of RGCS have reported empowerment as an outcome. A patient-reported outcome measure based on the concept of empowerment has been developed for use in clinical genetics services, with broad uptake internationally, including translation into a number of other languages and adaptation into a short-form version for ease of use [46–49]. Whilst adaptation to some items would be needed, this validated patient-reported outcome measure could be a valuable addition to future studies on RGCS. Primary qualitative research to elicit outcomes of importance, as planned as a component of the CODECS study, will also ensure that outcomes relevant to patients are included in future research.

Based on the number of qualitative studies included in this review, it is evident that researchers and clinicians are cognizant of the benefits of understanding patient experience and have appropriately used qualitative methods as an exploratory step to capture the patient perspective of RGCS. However, the translation of these exploratory findings into patient-centred outcomes that can be routinely incorporated into studies evaluating RGCS programs is needed. This review has identified a number of areas for future research, many of which will be addressed within the scope of the CODECS study. Stronger representation of the patient perspective is needed to ensure that RGCS is conducted in a people-centred manner. Public and patient involvement should be considered at the inception of research design, and researchers should strive to select patient-reported outcomes that have been developed using an evidence-base involving patients. Once complete, a COS will provide clear, evidence-based guidance for which outcomes should be measured as the starting point for all future studies of RGCS. Generalisability is also a consideration for future research. Compared to quantitative studies which were identified in 15 countries, qualitative data was only available for 6 countries. Future research should aim to incorporate international patient representation, or consider to what degree outcomes are likely to significantly differ across countries.

## Limitations

Publications not available in English were excluded due to a lack of resources for translation. The deductive method used to extract outcomes from qualitative studies holds some inherent limitations, including that the influence of the researcher(s) conducting the data extraction could alter the meaning within text excerpts due to unconscious knowledge or biases. This was recognized and minimized through the double coding and review of all coded excerpts, as well as grouping within outcome domains by a second reviewer and the wider study management group. Some limitations exist in the generalisability of the outcomes identified in this review. Qualitative studies included representation from 6 countries, predominantly of White/European populations, which is significantly less diverse than quantitative studies which included 15 countries. Within the 6 countries, demographics are further skewed towards high socioeconomic groups. Only 3 studies were in groups that had accessed expanded carrier screening panels, and as this is becoming increasingly the standard over small or ethnicity-based panels, further qualitative exploration in groups accessing large panels may be warranted to ensure all relevant outcomes are captured. Therefore, caution must be taken in assuming that the range of outcomes identified in this review would be generalizable to all populations. In accordance with the aims of the CODECS study, further work is underway to ensure that diverse patient perspectives are incorporated in the development of the core outcome set.

## Conclusion

This review identified outcomes that are important to people who access RGCS and will inform the development of a COS for population-based RGCS. We identified a number of outcomes that were not previously represented in quantitative studies, indicating that this review constitutes an important step in ensuring that the patient perspective is strongly represented in future stages of the CODECS study.

## Supplementary information

Supplementary Material
