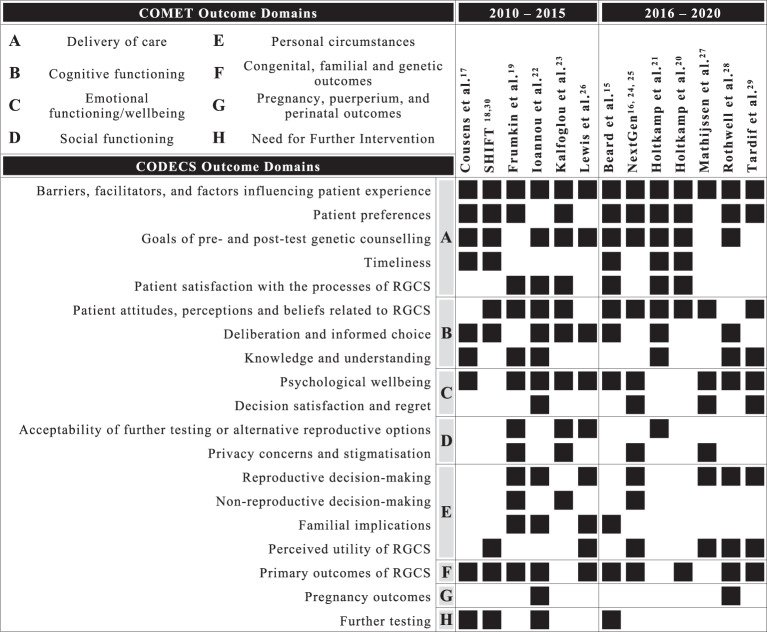# Correction: Incorporating patient perspectives in the development of a core outcome set for reproductive genetic carrier screening: a sequential systematic review

**DOI:** 10.1038/s41431-022-01099-6

**Published:** 2022-04-15

**Authors:** Ebony Richardson, Alison McEwen, Toby Newton-John, Ashley Crook, Chris Jacobs

**Affiliations:** Graduate School of Health, University of Technology Sydney, Sydney, NSW Australia

**Keywords:** Population screening, Genetic counselling, Genetic testing

Correction to: *European Journal of Human Genetics* 10.1038/s41431-022-01090-1, published online 28 March 2022

In Fig. 2 of this article, there is no references included; the figure should have appeared as shown below.