# Supplementary material for: Incorporating patient perspectives in the development of a core outcome set for reproductive genetic carrier screening: a sequential systematic review
Source: Eur J Hum Genet. 2022 Mar 28;30(7):756–65. doi: 10.1038/s41431-022-01090-1 (PMC9259674; doi:10.1038/s41431-022-01090-1)
Supplement: Supplementary file 1 — Supplementary Material [file 41431_2022_1090_MOESM1_ESM.pdf]

DESCRIPTION OF SUPPLEMENTAL DATA

Supplementary material 1 - Illustrative search strategy

Supplementary material 2 – Outcome domains with definitions and examples (new domains or updated domains indicated in blue)

Supplementary material 3 – Risk of bias assessment

Supplementary material 4 - Summary of included studies

SUPPLEMENTARY MATERIAL 1: Example Search Strategy (Medline Ovid)

REPRODUCTIVE GENETIC CARRIER SCREENING (RGCS		
**Keyword**	**#**	**Search Term**
Genetic Carrier Screening	1	Genetic Carrier Screening.mp<br>Genetic Carrier Screening/
Genetic Carrier Testing	2	Genetic Carrier Testing.mp
Carrier Screening	3	Carrier Screening.mp
Carrier Testing	4	Carrier Testing.mp
Preconception	5	Preconception*.mp
Expanded	6	Expanded.mp
Prenatal	7	Prenatal.mp
Reproductive	8	Reproduct*.mp
**RGCS Search**	**(1 or 2 or 3 or 4) AND (5 or 6 or 7 or 8) (2213 articles)**	

OUTCOME-RELATED TERMS		
**Keyword**	**#**	**Search Term**
Acceptability	1	Acceptability.mp<br>Patient acceptability of health care/
Attitude	2	Attitude*
Barrier	3	Barrier*.mp
Behaviour	4	Behavio*
Challenge	5	Challeng*.mp
Clinical utility	6	Clinical utility.mp
Decisional conflict	7	Decisional conflict.mp
Decision-making	8	Decision?making.mp<br>Decision Making/
Experience	9	Experience*.mp
Informed consent	10	Informed consent.mp<br>Informed Consent/
Knowledge	11	Knowledge.mp<br>Health Knowledge, Attitudes, Practice/
Outcomes	12	Outcome*.mp
Psychosocial	13	Psycho*.mp
Patient Satisfaction	14	Patient satisfaction.mp<br>Patient satisfaction/
Reproductive behaviour	15	Reproduct* behavio*.mp<br>Reproductive behaviour/
Understanding	16	Understand*.mp
Uptake	17	Uptake.mp
Willingness to pay	18	Willingness to pay.mp
**Outcomes Search**	**1-18 (OR) (7343415 articles)**	

METHODOLOGICAL TERMS		
**Keyword**	**#**	**Search Term**
Consensus	1	Consensus*.mp<br>Consensus/<br>Consensus Development Conference/
Feasibility		Feasib*.mp<br>Feasibility studies/
Focus group	2	Focus group*.mp<br>Focus groups/
Implementation		Implement*.mp
Interview	3	Interview*.mp<br>Interview/<br>Interviews as topic/
Mixed method	4	Mixed method*.mp
Pilot study	5	Pilot*.mp<br>Pilot Projects/
Program development or evaluation	6	Program*.mp<br>Program development/<br>Program evaluation/
Randomised control trial	7	Randomi?sed control* trial*.mp<br>Randomized Controlled Trials as Topic/
Survey/Questionnaire	8	Survey*.mp<br>“Surveys and Questionnaires”/<br>Health surveys/<br>Health care surveys/
Qualitative	9	Qualitative*.mp<br>Qualitative research/
Quantitative	10	Quantitative*.mp
**Methods Search**	**1-10 (OR) (2996983 articles)**	

“((Genetic Carrier Screening OR Genetic Carrier Testing OR Carrier Screening OR Carrier Testing) AND (Preconception* OR Expanded OR Prenatal OR Reproduct*)) **AND** ((Acceptab* OR Barrier OR Behavio* OR Challeng* OR Clinical Utility OR Decisional Conflict OR Decision?Making OR Experience* OR Informed Consent OR Knowledge OR Outcome* or Psycho* OR Patient Satisfaction OR Reproduct* Behavio* OR Understand* OR Uptake OR Willingness to Pay) **OR** (Consensus* OR Feasib* OR Focus Group* OR Implement* OR Interview* OR Mixed Method* OR Pilot* OR Program* OR Randomi?ed control* trial* OR Survey* OR Qualitative* OR Quantitative*))”

SUPPLEMENTARY MATERIAL 2: Outcome domains with examples

Core Area	Outcome Domain (COMET taxonomy)	Sub-domain (defined by SMG)	Definition	Example outcome
Physiological/ clinical	Congenital, familial and genetic outcomes	Primary outcomes of RGCS	Outcomes related to the results of RGCS	*- Carrier detection rate*<br>*- Identification of increased risk couples*
		Secondary or incidental outcomes of RGCS	Outcomes related to laboratory findings not related to the primary indication for screening. This may include findings related to variants of uncertain significance, incidental findings (defined here are as findings that were unexpected), and secondary findings (defined here are secondary outcomes that were deliberately looked for and therefore are not truly incidental)	*- Identification of variants of uncertain significance*<br>*- Identification of homozygous, hemizygous or compound heterozygous individuals at risk for developing one of the screened conditions*
		Other laboratory outcomes	Outcomes related to additional laboratory outcomes other than the primary results	*- Rate of test failure due to insufficient DNA in patient sample*<br>*- Rate of false-positive screening via biochemical analysis*
	Pregnancy, puerperium and perinatal outcomes	Postnatal outcomes	Outcomes related to new affected cases of the condition being screened	*- Number of individuals born with the condition(s) being screened for*<br>*- Reasons for new affected births*
		Pregnancy outcomes	Outcomes related to the impact of screening results on future pregnancies	*- Results of prenatal diagnosis in future pregnancies*<br>*- Decision to continue or terminate affected foetuses in future pregnancies*<br>*- Results of prenatal diagnosis in pregnancies at the time of screening*<br>*- Decision to continue or terminate an affected fetus in current pregnancy*
Life Impact	Cognitive functioning	Patient attitudes, perceptions and beliefs related to RGCS	Outcomes related to patient's attitudes, perceptions or beliefs about RGCS	*- Perception that RGCS would alter reproductive decisions*<br>*- Attitude regarding recommending carrier screening to others*
		Deliberation and informed choice	Outcomes related to making an informed choice to undertake RGCS	*- Deliberation on the decision to accept or decline testing*<br>*- Informed choice (congruence of attitudes, knowledge and test uptake)*
		Knowledge and understanding	Outcomes related to knowledge, incorporating concepts of understanding, recall and retention.	*- Understanding of the information received during genetic counselling session*<br>*- Knowledge before and after pre-test genetic counselling*
	Delivery of care	Intention and uptake	Outcomes related to actual or intention to uptake an offer of RGCS	*- Uptake of RGCS*<br>*- Intention to accept the offer of RGCS*
		Barriers, facilitators and factors influencing patient experience	Outcomes related to reasons for and against uptake of services, including offers of RGCS and further testing, as well as factors that influence experience of these services	*- Reasons for accepting/declining an offer of RGCS*<br>*- Reasons or factors related to emotional reactions and psychological wellbeing*<br>*- Sources of additional information used for decision-making regarding uptake of RGCS*
		Genetic counselling resource use	Outcomes related to the use and conduct of genetic counselling services	*- Number of post-test genetic counselling consultations*<br>*- Time required for pre-test genetic counselling session*
		Goals of pre- and post-test genetic counselling	Outcomes related to the patient experience of pre- and post-test interactions with their health providers	*- Genetic counselling supported informed decision-making*<br>*- Timing and method of information provision promoted understanding*
		Practice guidelines/recommendations	Outcomes related to clinical practice recommendations	*- Ordering clinicians selection of conditions according to practice recommendations*
		Patient preferences	Outcomes related to patient preferences regarding the offer of RGCS	*- Preference regarding individual or couple-based results*<br>*- Preference regarding conditions included in RGCS*

		Patient satisfaction with the processes of RGCS	Outcomes related to patient satisfaction with services related to RGCS	*- Satisfaction with pre-test genetic counselling*
		Timeliness	Outcomes related to the timeliness of delivery of care in RGCS programs	*- Mean gestational age at time of reproductive carrier screening*<br>*- Offer of reproductive carrier screening to women before 10 weeks gestation*
	Emotional functioning/wellbeing	Decision satisfaction and regret	Outcomes related to decisional satisfaction or regret at a later timepoint	*- Distress or remorse after a healthcare decision measured at a later timepoint*<br>*- Satisfaction with the decision to accept/decline screening*
		Psychological wellbeing	Outcomes related to the psychological impact of RGCS	*- Anxiety (measured at a range of timepoints)*<br>*- Subjective distress associated with being identified as a heterozygote, at a later timepoint*
	Perceived health status	Perception of personal health status after RGCS	Outcomes related to the impact of RGCS on perception of personal health	*- Impact of results on perception of own health*
	Personal circumstances	Decision-making (non-reproductive)	Outcomes related to the impact of results on decisions other than reproductive planning	*- Impact of negative result on decisions related to insurance, healthcare and lifestyle*<br>*- Number of prospective marriages cancelled due to identification as an increased risk couple (pre-marital screening programs)*
		Decision-making (reproductive)	Outcomes related to impact of results on decision-making for reproductive planning, including perceived or actual impact on these decisions	*- Pursued or planned to pursue alternate reproductive options*<br>*- Intended reproductive decisions if identified as an increased risk couple*
		Familial implications	Outcomes related to the impact of results of patient relationships	*- Impact of results on couple's relationship*<br>*- Number of heterozygotes that informed family members of their results*
		Perceived utility of RGCS	Outcomes related to patient's perceptions of the impact of RGCS and how they utilised the results	*- Confidence or empowerment related to reproductive decision-making*<br>*- Results were available in a timely manner that allowed for consideration and decision-making*
	Social functioning	Acceptability of further testing or alternative reproductive options	Outcomes related to patients' perspectives on prenatal diagnosis, termination of pregnancy, and preimplantation genetic diagnosis	*- Religious views on PND, PGD and TOP*<br>*- Patient perceptions of practical difficulties of IVF and PGD*
		Privacy concerns and stigmatisation	Outcomes related to the impact of results on privacy and stigmatisation	*- Concern regarding privacy or confidentiality*<br>*- Fear of discrimination by insurance companies*
Resource Use	Need for further intervention	Further testing	Outcomes related to the use of further testing for various purposes including clarifying reproductive risk as a couple, testing during a pregnancy, or electing PGD	*- Uptake of partner testing*<br>*-Uptake of prenatal diagnosis in increased risk pregnancies at the time of screening*<br>*- Uptake of postnatal diagnostic testing in decliners of prenatal diagnosis in a current pregnancy at the time of screening*<br>*- Uptake of preimplantation genetic diagnosis in increased risk couples*

Documented updates to CODECS domains (indicated in blue):

"Genetic counselling" was changed to "Genetic counselling resource use", "Knowledge" was changed to "Knowledge and understanding", "Barrier and facilitators" was changed to "Barriers, facilitators, and factors influencing patient experience", "Attitudes and perceptions" was changed to "Patient attitudes, perceptions and beliefs related to RGCS", "Patient satisfaction" was changed to "Patient satisfaction with the processes of RGCS", "Information sources" was merged with "Barriers, facilitators, and factors influencing patient experience"

SUPPLEMENTARY MATERIAL 3: QualSyst Risk of Bias Assessment. Higher scores represent higher quality and less risk of bias

First Author and Year	QualSyst Score
Beard (2016)[1]	0.75
Clarke (2018)[2]	0.6
Cousens (2013)[3]	0.8
Dormandy (2010)[4]	0.6
Frumkin (2011)[5]	0.45
Holtkamp (2018) [6]	0.8
Holtkamp (2019) [7]	0.7
Ioannou (2015)[8]	0.75
Kalfoglou (2011)[9]	0.6
Kraft (2018)_1[10]	0.5
Kraft (2018)_2[11]	0.55
Lewis (2012)[12]	0.85
Mathijssen (2018)[13]	0.6
Rothwell (2017)[14]	0.7
Tardif (2018)[15]	0.75
Tsianakas (2012)[16]	0.7

SUPPLEMENTARY MATERIAL 4: Summary of included studies

Study ID	Author and Year	Publication Title	Country	Test Type	Study Design	Sequential Review Status	Number of Outcomes
1	Ioannou et al. 2015[8]	"Suddenly having two positive people who are carriers is a whole new thing" - experiences of couples both identified as carriers of cystic fibrosis through a population-based carrier screening program in australia	Australia	CF only	Mixed Methods<br>Associated publications from quantitative review[17,18]	Qualitative review only	32
2	Cousens et al. 2013[3]	"He didn't say that thalassaemia might come up" - beta-thalassaemia carriers' experiences and attitudes	Australia	Haemoglobinopathies	Qualitative only	Qualitative review only	17
3	Beard et al. 2016[1]	"I'm healthy, it's not going to be me": exploring experiences of carriers identified through a population reproductive genetic carrier screening panel in australia	Australia	3-gene	Qualitative only	Qualitative review only	24
4	Tardif et al. 2018[15]	Experience of carrier couples identified through a population-based carrier screening pilot program for four founder autosomal recessive diseases in saguenay-lac-saint-jean	Canada	Founder	Qualitative only	Qualitative review only	14
5	Frumkin et al. 2011[5]	"The most important test you'll ever take": attitudes toward confidential carrier matching and open individual testing among modern-religious jews in israel	Israel	Founder	Qualitative only	Qualitative review only	14
6	Holtkamp et al. 2019[7]	Direct-to-consumer carrier screening for cystic fibrosis via a hospital website: a 6-year evaluation	The Netherlands	CF only	Qualitative only	Qualitative review only	8
7	Mathijssen et al. 2018[13]	Preconception carrier screening for multiple disorders: evaluation of a screening offer in a dutch founder population	The Netherlands	Founder	Mixed Methods<br>Associated publications from quantitative review[19]	Included in both reviews	9

8	Holtkamp et al. 2018[6]	Experiences of a high-risk population with prenatal hemoglobinopathy carrier screening in a primary care setting: a qualitative study	The Netherlands	Haemoglobinopathies	Qualitative only	Qualitative review only	15
9	Dormandy et al. 2010[4]	Antenatal screening for haemoglobinopathies in primary care: a cohort study and cluster randomised trial to inform a simulation model. The screening for haemoglobinopathies in first trimester (shift) trial	UK	Haemoglobinopathies	Mixed Methods Publications from quantitative review[20,21]	Included in both reviews	17
	Tsianakas et al. 2012[16]	Offering antenatal sickle cell and thalassaemia screening to pregnant women in primary care: a qualitative study of women's experiences and expectations of participation	UK	Haemoglobinopathies	Mixed Methods Publications from quantitative review[20,21]	Qualitative review only	
10	Lewis et al. 2012[12]	Reproductive empowerment: the main motivator and outcome of carrier testing	UK	Unspecified	Qualitative only	Qualitative review only	11
11	Clarke et al. 2018[2]	Assessment of willingness to pay for expanded carrier screening among women and couples undergoing preconception carrier screening	USA	ECS	Mixed Methods Publications from quantitative review[22,23]	Included in both reviews	18
	Kraft et al. 2018[10]	Patient actions and reactions after receiving negative results from expanded carrier screening	USA	ECS	Mixed Methods Publications from quantitative review[22,23]	Included in both reviews	
	Kraft et al. 2018[11]	Patient perspectives on the use of categories of conditions for decision making about genomic carrier screening results	USA	ECS	Mixed Methods Publications from quantitative review[22,23]	Qualitative review only	
12	Rothwell et al. 2017[14]	Experiences among Women with Positive Prenatal Expanded Carrier Screening Results	USA	ECS	Qualitative only	Qualitative review only	17
13	Kalfoglou et al. 2011[9]	Orthodox Ashkenazi Young Adults' Knowledge, Experiences, Attitudes, and Beliefs About Genetic Carrier Testing	USA	Founder	Qualitative only	Qualitative review only	11

References:

1. Beard CA, Amor DJ, Di Pietro L, Archibald AD. "I'm Healthy, It's Not Going To Be Me": Exploring experiences of carriers identified through a population reproductive genetic carrier screening panel in Australia. *Am J Med Genet A.* 2016;170(8):2052-2059.
2. Clarke EV, Schneider JL, Lynch F, et al. Assessment of willingness to pay for expanded carrier screening among women and couples undergoing preconception carrier screening. *PLoS One.* 2018;13(7):e0200139.
3. Cousens NE, Gaff CL, Metcalfe SA, Delatycki MB. "He didn't say that thalassaemia might come up" - beta-thalassaemia carriers' experiences and attitudes. *J Community Genet.* 2013;4(2):223-232.
4. Dormandy E, Bryan S, Gulliford MC, et al. Antenatal screening for haemoglobinopathies in primary care: a cohort study and cluster randomised trial to inform a simulation model. The Screening for Haemoglobinopathies in First Trimester (SHIFT) trial. *Health Technol Assess.* 2010;14(20):1-160.
5. Frumkin A, Raz AE, Plesser-Duvdevani M, Lieberman S. "The Most Important Test You'll Ever Take"?: attitudes toward confidential carrier matching and open individual testing among modern-religious Jews in Israel. *Soc Sci Med.* 2011;73(12):1741-1747.
6. Holtkamp KCA, Lakeman P, Hader H, et al. Experiences of a High-Risk Population with Prenatal Hemoglobinopathy Carrier Screening in a Primary Care Setting: a Qualitative Study. *J Genet Couns.* 2018;27(3):635-646.
7. Holtkamp KCA, Henneman L, Gille JJP, Meijers-Heijboer H, Cornel MC, Lakeman P. Direct-to-consumer carrier screening for cystic fibrosis via a hospital website: a 6-year evaluation. *J Community Genet.* 2019;10(2):249-257.
8. Ioannou L, Delatycki MB, Massie J, Hodgson J, Lewis S. "Suddenly Having two Positive People who are Carriers is a Whole New Thing" - Experiences of Couples Both Identified as Carriers of Cystic Fibrosis Through a Population-Based Carrier Screening Program in Australia. *J Genet Couns.* 2015;24(6):987-1000.
9. Kalfoglou AL, Broder M. Orthodox Ashkenazi Young Adults' Knowledge, Experiences, Attitudes, and Beliefs About Genetic Carrier Testing. *AJOB Primary Research.* 2011;2(2):1-7.
10. Kraft SA, Schneider JL, Leo MC, et al. Patient actions and reactions after receiving negative results from expanded carrier screening. *Clin Genet.* 2018;93(5):962-971.
11. Kraft SA, McMullen CK, Porter KM, et al. Patient perspectives on the use of categories of conditions for decision making about genomic carrier screening results. *Am J Med Genet A.* 2018;176(2):376-385.
12. Lewis C, Skirton H, Jones R. Reproductive empowerment: the main motivator and outcome of carrier testing. *J Health Psychol.* 2012;17(4):567-578.
13. Mathijssen IB, Holtkamp KCA, Ottenheim CPE, et al. Preconception carrier screening for multiple disorders: evaluation of a screening offer in a Dutch founder population. *Eur J Hum Genet.* 2018;26(2):166-175.
14. Rothwell E, Johnson E, Mathiesen A, et al. Experiences among Women with Positive Prenatal Expanded Carrier Screening Results. *J Genet Couns.* 2017;26(4):690-696.
15. Tardif J, Pratte A, Laberge AM. Experience of carrier couples identified through a population-based carrier screening pilot program for four founder autosomal recessive diseases in Saguenay-Lac-Saint-Jean. *Prenat Diagn.* 2018;38(1):67-74.

16. Tsianakas V, Atkin K, Calnan MW, Dormandy E, Marteau TM. Offering antenatal sickle cell and thalassaemia screening to pregnant women in primary care: a qualitative study of women's experiences and expectations of participation. *Health Expect.* 2012;15(2):115-125.
17. Ioannou L, Massie J, Collins V, McClaren B, Delatycki MB. Population-based genetic screening for cystic fibrosis: attitudes and outcomes. *Public Health Genomics.* 2010;13(7-8):449-456.
18. Ioannou L, Massie J, Lewis S, McClaren B, Collins V, Delatycki MB. 'No thanks'-reasons why pregnant women declined an offer of cystic fibrosis carrier screening. *J Community Genet.* 2014;5(2):109-117.
19. Mathijssen IB, Henneman L, van Eeten-Nijman JM, et al. Targeted carrier screening for four recessive disorders: high detection rate within a founder population. *Eur J Med Genet.* 2015;58(3):123-128.
20. Brown K, Dormandy E, Reid E, Gulliford M, Marteau T. Impact on informed choice of offering antenatal sickle cell and thalassaemia screening in primary care: a randomized trial. *J Med Screen.* 2011;18(2):65-75.
21. Dormandy E, Gulliford M, Bryan S, et al. Effectiveness of earlier antenatal screening for sickle cell disease and thalassaemia in primary care: cluster randomised trial. *BMJ.* 2010;341:c5132.
22. Gilmore MJ, Schneider J, Davis JV, et al. Reasons for declining preconception expanded carrier screening using genome sequencing. *J Genet Couns.* 2017;26(5):971-979.
23. Punj S, Akkari Y, Huang J, et al. Preconception carrier screening by genome sequencing: results from the clinical laboratory. *Am J Hum Genet.* 2018;102(6):1078-1089.
